# Colposcopy Accuracy and Diagnostic Performance: A Quality Control and Quality Assurance Survey in Italian Tertiary-Level Teaching and Academic Institutions—The Italian Society of Colposcopy and Cervico-Vaginal Pathology (SICPCV)

**DOI:** 10.3390/diagnostics13111906

**Published:** 2023-05-29

**Authors:** Massimo Origoni, Francesco Cantatore, Francesco Sopracordevole, Nicolò Clemente, Arsenio Spinillo, Barbara Gardella, Rosa De Vincenzo, Caterina Ricci, Fabio Landoni, Maria Letizia Di Meo, Andrea Ciavattini, Jacopo Di Giuseppe, Eleonora Preti, Anna Daniela Iacobone, Carmine Carriero, Miriam Dellino, Massimo Capodanno, Antonino Perino, Cesare Miglioli, Luca Insolia, Maggiorino Barbero, Massimo Candiani

**Affiliations:** 1 Department of Obstetrics & Gynecology, IRCCS Ospedale San Raffaele, Vita Salute San Raffaele University School of Medicine, 20132 Milan, Italy; francesco.cantatore@hsr.it (F.C.); massimo.candiani@hsr.it (M.C.); 2 Gynecological Oncology Unit, IRCCS Oncological Referral Center (CRO), National Cancer Institute, 33081 Aviano, Italy; fsopracordevole@cro.it (F.S.); nicolo.clemente@cro.it (N.C.); 3 Department of Obstetrics & Gynecology, IRCCS Policlinico San Matteo, University of Pavia, 27100 Pavia, Italy; a.spinillo@smatteo.pv.it (A.S.); barbara.gardella@gmail.com (B.G.); 4 Gynecological Oncology Unit, Department of Woman and Child Health and Public Health, IRCCS Policlinico Universitario A. Gemelli, 00168 Rome, Italy; rosa.devincenzo@unicatt.it; 5 Department of Health Sciences and Public Health, Catholic University of the Sacred Hearth, 00168 Rome, Italy; caterina.ricci@policlinicogemelli.it; 6 Department of Medicine and Surgery, University of Milano Bicocca, Clinic of Obstetrics and Gynecology, IRCCS San Gerardo dei Tintori, 20900 Monza, Italy; fabio.landoni@unimib.it (F.L.); marialetizia.dimeo@gmail.com (M.L.D.M.); 7 Gynecologic Section, Department of Odontostomatological and Specialized Clinical Sciences, Marche Polytechnic University, 60123 Ancona, Italy; a.ciavattini@staff.univpm.it (A.C.); jacopo.digiuseppe@ospedaliriuniti.marche.it (J.D.G.); 8 Preventive Gynecology Unit, IRCCS European Institute of Oncology (IEO), 20141 Milan, Italy; eleonora.preti@ieo.it (E.P.); annadaniela.iacobone@ieo.it (A.D.I.); 9 Department of Biomedical Sciences, University of Sassari, 07100 Sassari, Italy; 10 Interdisciplinary Department of Medicine, University of Bari Aldo Moro, 70121 Bari, Italy; carmine.carriero@uniba.it (C.C.); miriam.dellino@uniba.it (M.D.); 11 Department of Obstetrics and Gynecology, University of Napoli, 80138 Naples, Italy; massimocapodanno@virgilio.it; 12 Department of Obstetrics and Gynecology, University of Palermo, 90146 Palermo, Italy; antonio.perino@unipa.it; 13 Research Center for Statistics, University of Geneva, 1201 Geneva, Switzerland; cesare.miglioli@unige.ch (C.M.); luca.insolia@unige.ch (L.I.); 14 Department of Obstetrics and Gynecology, Azienda Sanitaria Locale di Asti, 14100 Asti, Italy; barberom@tin.it

**Keywords:** colposcopy, QC, QA, colposcopy sensitivity, diagnostic accuracy, cervical cancer prevention, CIN, SIL, colposcopy standards

## Abstract

Quality Control (QC) and Quality Assurance (QA) principles are essential for effective cervical cancer prevention. Being a crucial diagnostic step, colposcopy’s sensitivity and specificity improvements are strongly advocated worldwide since inter- and intra-observer differences are the main limiting factors. The objective of the present study was the evaluation of colposcopy accuracy through the results of a QC/QA assessment from a survey in Italian tertiary-level academic and teaching hospitals. A web-based, user-friendly platform based on 100 colposcopic digital images was forwarded to colposcopists with different levels of experience. Seventy-three participants were asked to identify colposcopic patterns, provide personal impressions, and indicate the correct clinical practice. The data were correlated with a panel of experts’ evaluation and with the clinical/pathological data of the cases. Overall sensitivity and specificity with the threshold of CIN2+ accounted for 73.7% and 87.7%, respectively, with minor differences between senior and junior candidates. Identification and interpretation of colposcopic patterns showed full agreement with the experts’ panel, ranging from 50% to 82%, in some instances with better results from junior colposcopists. Colposcopic impressions correlated with a 20% underestimation of CIN2+ lesions, with no differences linked to level of experience. Our results demonstrate the good diagnostic performance of colposcopy and the need for improving accuracy through QC assessments and adhesion to standard requirements and recommendations.

## 1. Introduction

Colposcopy represents the recommended second-level procedure for the assessment of the uterine cervix as part of a cervical cancer screening program; it is indicated following the detection of primary test positivity according to specific guidelines, and its main objective is the early detection of high-grade cervical intraepithelial neoplasia (CIN2+) [1,2]. Colposcopic observation thus relies on the visual interpretation of macroscopic changes in color and morphology of the genital mucosae and on the correlation of specific patterns with different degrees of cervical disease. According to this intrinsic aspect of the procedure, colposcopy carries the cost of significant observer-dependent performance and thus the risk of lacking sensitivity and accuracy.

The performance of the exam is fundamental and mainly depends upon three steps: the identification of the squamocolumnar junction (SCJ), the correct assessment of the Transformation Zone (TZ) and the decision to take a biopsy/biopsies in the most appropriate cervical area.

Although colposcopy plays a fundamental role in the prevention of cervical cancer as it allows the identification, treatment, and/or follow-up of pre-cancer lesions, the accuracy of the procedure is largely influenced by a high degree of subjectivity and low reproducibility. This may lead to high rates of severe lesions under diagnosis or even cancer under detection. In this view, Artificial Intelligence (AI) may represent a promising option to overcome this limitation.

Colposcopy performance has been largely investigated and reported in different settings and different geographic areas [3,4,5]; almost all published data are consistent in reporting a large variability in terms of both sensitivity and specificity, with values ranging from 30% to 90% and from 40% to 95%, respectively. In this view, the colposcopic impression (CI), based on the detailed identification and interpretation of the different aspects of the TZ, represents the major issue, being closely correlated with the operator’s decision to perform a targeted biopsy [6,7] and the success of the cervical cancer prevention strategy.

In the last few years, the application of Quality Control (QC) and Quality Assurance (QA) principles to assess the accuracy and performance of colposcopy has been advocated as of pivotal importance and is a strong recommendation worldwide [8,9,10,11,12,13]. 

The present study aims, through the multicentric involvement of major Italian teaching and academic gynecological institutions, to investigate the accuracy and quality assessment of colposcopy and, consequently, to determine the performance of operators with different levels of expertise in the field. In particular, the study was designed to assess the probability for a patient with a histologically confirmed cervical lesion of being incorrectly managed through the colposcopic workup (e.g., under detection of significant TZ alterations, not having a biopsy performed, or having a biopsy in an incorrect site). The secondary objective of the study was the development of a user-friendly online platform where Quality Control of colposcopy could be easily achieved and that could potentially be proposed and promoted for a nationwide QC and QA program.

## 2. Materials and Methods

One hundred (n. 100) colposcopic digital images were selected by a panel of experts among a large database of clinical cases with a comprehensive dataset of patients’ demographic information, clinical history, cytological, virological (HPV-DNA detection), and pathological data. In particular, 35 were histologically negative (or without any type of lesion), 34 were low-grade lesions (HPV or CIN1), 24 were high-grade lesions (CIN2, CIN3, or in situ carcinoma), and 7 were pathologically proven invasive squamous or adenocarcinoma.

**Figure 1:**
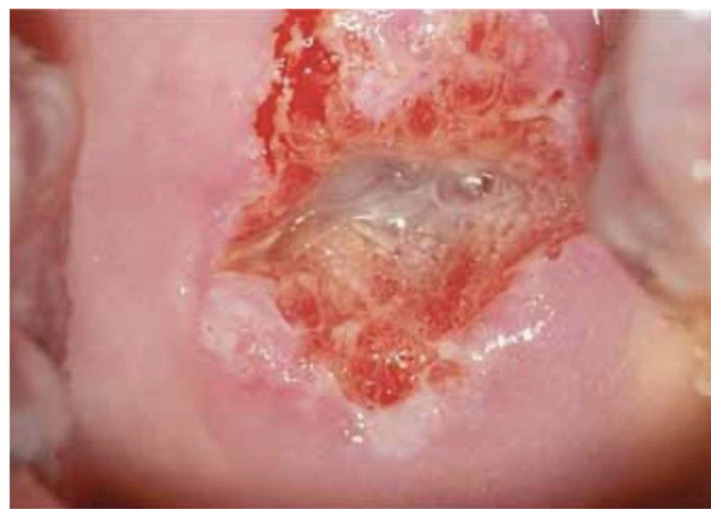
*Fully visible* SCJ—G2—biopsy indicated.

**Figure 2:**
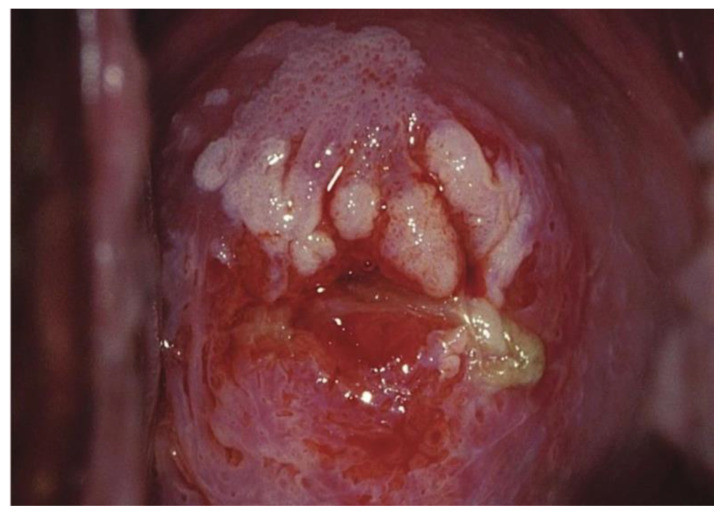
*Fully visible* SCJ—G2—biopsy indicated.

**Figure 3:**
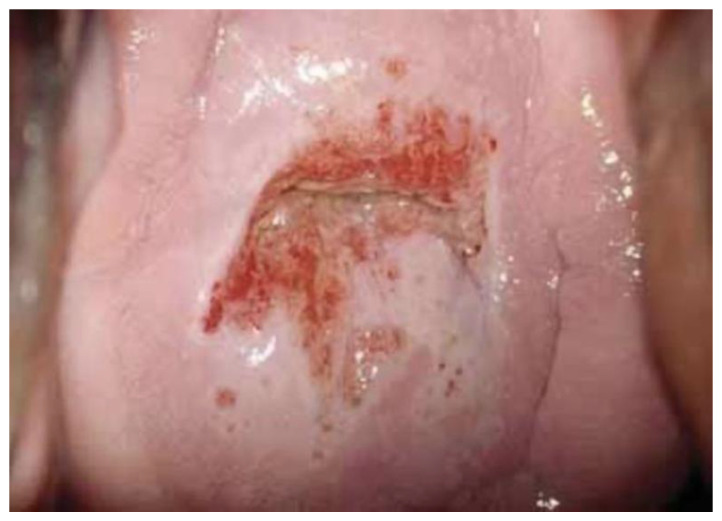
*Fully visible* SCJ—G2—biopsy indicated.

Images were deliberately identified when an objectively “difficult” colposcopic pattern was present. Nevertheless, the quality and resolution of all images, complete visibility of the entire cervix, absence of mucus/blood, and good representation of normal/abnormal colposcopic patterns were always identifiable; randomly selected images are illustrated as examples in Figure 1, Figure 2 and Figure 3.

The experts’ panel, for each single case, identified and recorded the following items: (1) assessment of colposcopic patterns according to the 2011 International Federation of Cervical Pathology and Colposcopy (IFCPC) nomenclature [14] and the 2017 American Society of Colposcopy and Cervical Pathology (ASCCP) terminology proposal [15]; (2) colposcopic impression, categorized as (2.1) negative, (2.2) favour low-grade lesion (Human Papillomavirus infection—Cervical Intraepithelial Neoplasia grade 1 CIN1), (2.3) favor high-grade lesion (Cervical Intraepithelial Neoplasia grade 2–3 CIN2+ or in situ squamous/adenocarcinoma), (2.4) favor malignant lesion (invasive squamous carcinoma or adenocarcinoma); (3) indication for taking a single biopsy or up to a maximum of 3 biopsies; and (4) the most appropriate area to be biopsied. 

By the use of Qualtrix XM^®^ software (2022 version) (www.qualtrics.com), an online platform was developed, either loggable via personal computers, tablets, or smartphones; following log-in, the application delivered the colposcopic digital high-resolution images integrated by a caption with details about the patient’s age and primary screening results (cervical cytology and/or HPV-DNA detection), and a set of questions focused on: (1) squamocolumnar junction (SCJ) interpretation; (2) Transformation Zone (TZ) assessment; (3) biopsy indication; (4) areas suitable for performing biopsy; and (5) colposcopic impression.

The web link to the platform was forwarded to 10 academic and teaching Ob/Gyn Italian institutions, all having tertiary-level preventive oncological gynecology units, inviting colposcopy operators to anonymously attend the survey, detailing their respective level of expertise (<5 years vs. >5 years of colposcopy practice). Almost all juniors were residents/fellows of the participating institutions. The workload to complete the exam was anticipated to be at least 90 min according to the survey’s characteristics, and it had to be finished in a single slot; at the end, each participant was provided with a final score but was not informed of the rate of correct/incorrect answers or the specification of the correct/incorrect ones. After completion of the test, the same could not be performed again because the platform credentials were no longer valid to log in to the application. 

Data were collected, centralized, and recorded by the promoting investigators and analyzed using the R statistical software (www.r-project.org); participants responses to the test were compared with those of the committee and analyzed with those of variables treated as categorical. Pearson’s chi-squared test (with Yates’ continuity correction) and Cohen’s *kappa* coefficient of agreement (95% CI intervals) were used to estimate the strength of associations; a *p* value < 0.05 was considered statistically significant, with *kappa* 0.60–0.80 indicating substantial agreement among observers [16,17]. The study design, methodology, and results were approved by the Scientific Committee of the Italian Society of Colposcopy and Cervico-Vaginal Pathology (SICPCV).

## 3. Results

The survey was conducted between January and April 2022 with the participation of 10 Italian centers: seventy-three (n. 73) colposcopists logged in to the web platform, 56 (76.7%) of them completing the whole test, and 17 (23.3%) only partially. The mean completion rate of the test for this latter subgroup of participants was 49%. The overall number of colposcopic observations/interpretations accounted for a total of 6155, upon which the survey has been performed. According to the level of colposcopic experience and practice, 27 (37%) participants reported a < 5 year practice in colposcopy (juniors) and 46 (63%) a personal experience > 5 years (seniors). No data were available regarding the number/year of colposcopies performed by participants.

The first part of the results analysis was primarily targeted at the identification of some intrinsic features of colposcopy, with the aim of evaluating the diagnostic accuracy and QC of the second-level colposcopy-based cervical cancer prevention workup. The overall analysis of the survey data in terms of colposcopy accuracy provided sensitivity and specificity rates of 61.6% and 77.1%, respectively; according to colposcopists’ experience, sensitivity was 60.6% for seniors and 62.0% for juniors, while specificity was 76.7% and 77.4%, respectively. Considering the histology threshold of CIN2+, specificity increased to 87.7% (seniors 86.2% vs. juniors 88.6%).

In details, sensitivity increased from 60.9% in low-grade cases (HPV or CIN1) to 73.7% in high-grade cases (CIN2+); no statistically significant differences were obtained comparing seniors vs. juniors’ rates of sensitivity (Table 1).

Despite lacking statistical significance, senior colposcopists sensitivity was always inferior compared to juniors, with the only exception of CIN2-CIN3 cases (64.9% vs. 62.3%); when cancer cases were added to CIN2-CIN3 in a single analysis, the sensitivity rates of the two subgroups of colposcopists were closely comparable (73.5% vs. 73.9%). As for specificity, juniors’ performance was again superior.

Table 2 shows the results according to the squamocolumnar junction (SCJ) evaluation, with the adoption of the 2011 IFCPC terminology [14]. Full agreement with the experts’ panel was recorded in 81.2% when a *fully visible* SCJ was present, in 51.4% in *not fully visible* SCJ cases, and in 64.9% in *not visible* SCJ cases. Comparing seniors with juniors, a significant statistical difference was observed in *not visible* SCJ cases only (67.5% vs. 60.7%; *p* = 0.011). The Cohen’s *kappa* correlation coefficient accounted for 0.49 (95% CI: 0.47–0.51) when the entire group of colposcopists was considered, for 0.49 (95% CI: 0.47–0.52) in the seniors group, and for 0.48 (95% CI: 0.45–0.51) in junior colposcopists. The highest rate of incorrect SCJ interpretation was recorded within the *not fully visible* SCJ group, where it accounted for 48.6%, with no statistical difference between seniors and juniors (48.1% vs. 49.5%).

The same analysis was performed adopting the SCJ nomenclature proposal suggested by the American Society of Cervical Pathology and Colposcopy in 2017 [15], which divided the SCJ into two colposcopic categories only: *fully visible* and *not fully visible*. Full agreement with the experts increased to 75% in the *not fully visible* SCJ subgroup, with a statistically significant difference between seniors and juniors (77.1% vs. 72.8%, respectively; *p* = 0.011). The Cohen’s *kappa* concordance coefficient also increased from 0.49 to 0.57 (95% CI: 0.54–0.59) for the whole set of participants, from 0.49 to 0.57 (95% CI: 0.55–0.60) for the seniors, and from 0.48 to 0.56 (95% CI: 0.52–0.59) for the juniors group. Table 3 summarizes these results.

Table 4 shows the results regarding colposcopists’ interpretation of the Transformation Zone (TZ) compared with the experts’ panel. 

Full agreement was observed in 73.2% of Type 1, 53.8% of Type 2, and 66.7% of Type 3 TZ cases; within each group of TZ, a statistically significant difference was demonstrated comparing seniors to juniors: in particular, Type 1 and Type 2 TZ were better identified by junior colposcopists (79% vs. 69.5% and 55.9% vs. 52.3%, respectively; *p* < 0.05), while Type 3 TZ was significantly better identified by seniors (71.7% vs. 58.3%; *p* < 0.05). 

In this analysis, the highest rate of incorrect interpretation was identified in senior colposcopists evaluating Type 2 TZ cases (47.7%), while the lowest rate was recorded in juniors’ evaluation of Type 1 TZ (21%).

The second part of the survey results analysis was conversely targeted to investigate the accuracy of the colposcopic procedure through the assessment of colposcopic interpretation of cervical patterns and its influence on the operators’ clinical decisions. 

As far as it concerned the assessment of grade (G) of the colposcopic pattern compared to proven histology, the following results were obtained: full agreement with histology was achieved in 60.59% of cases with G1/low-grade lesions, in 59.11% of G2/high-grade lesions, and in 64.64% of colposcopic patterns suspicious for cancer and histologically confirmed cervical malignancy; these concordance rates can also be seen as PPV of colposcopy. 

Interestingly, 5.05% and 19.26% of cases with a histologically proven CIN2+ were categorized as colposcopically negative or G1 by participants, respectively. 

On the other hand, overestimation of the colposcopic pattern reached the highest rate in histologically proven low-grade lesions (HPV-CIN1), which were classified as G2 in 24.70% of cases (Table 5).

A similar analysis was performed considering the colposcopic impression formulated by colposcopists compared to histology. 

A negative colposcopic impression correlated with a negative histology in 77.9% of cases, allowing this figure to be seen as NPV. Taking into consideration histologically confirmed high-grade lesions (CIN2-CIN3), which represent the main objective of the cervical cancer prevention strategy, the colposcopic impression of a high-grade lesion was correctly formulated by colposcopists in 59.4% of cases. 

When cancer cases were added to CIN2/CIN3, the PPV of a high-grade lesion colposcopic impression increased to 70.5%. 

The PPV of a colposcopic impression suspicious for cancer was 64.4% (*p* < 0.05; Cohen’s *kappa* correlation coefficient = 0.51; 95% CI: [0.50–0.53]) (Table 6).

**Figure 4:**
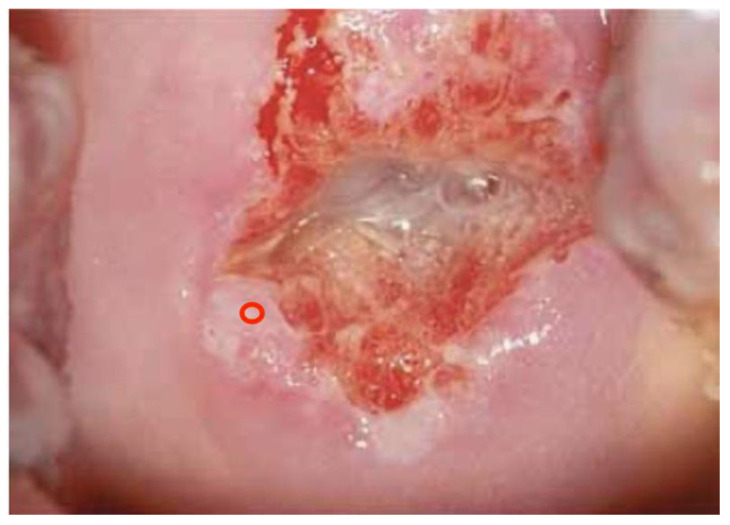
A single biopsy is indicated (correct site).

**Figure 5:**
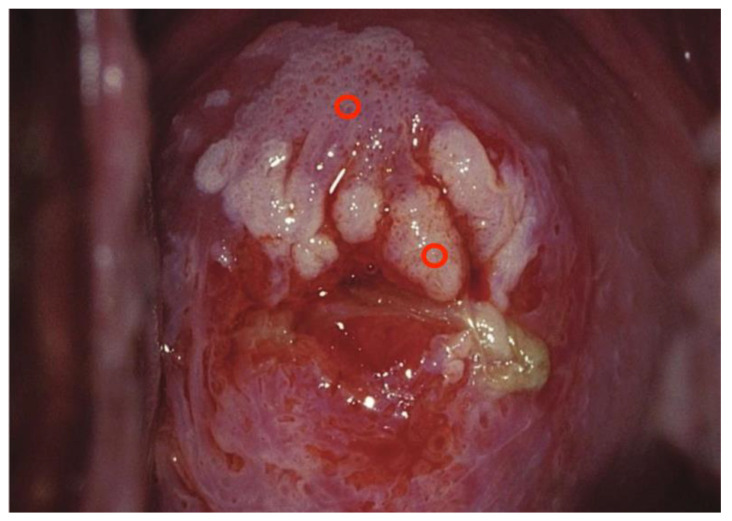
Multiple biopsies indicated (correct sites).

**Figure 6:**
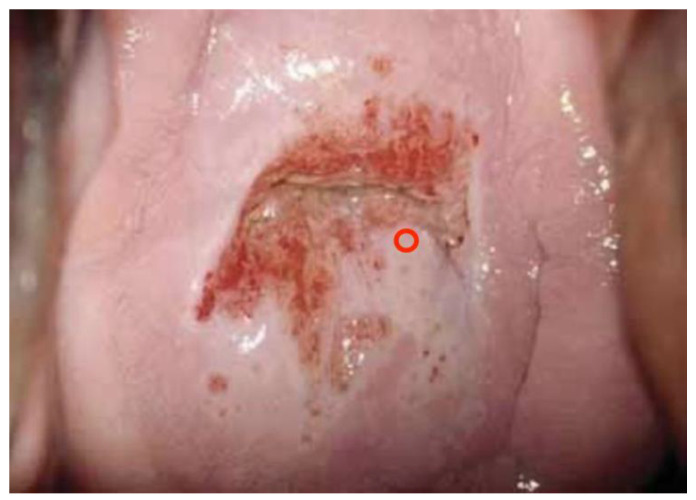
A single biopsy is indicated (correct site).

Directly correlated with the colposcopic impression and the G assessments, colposcopists were asked to indicate the need for taking biopsy/biopsies and the cervical site they thought was the most appropriate for histological confirmation; biopsies were performed in 3404 cases out of 6155 in the case of the experts panel (55%), and in 3482 cases out of 6155 (56%) in the case of candidates. Figure 4, Figure 5 and Figure 6 illustrate how the biopsy/biopsies sites were indicated by colposcopists.

According to colposcopists experience, junior colposcopists performed biopsies in 52.7% of the whole set of cases, while more experienced operators performed them in 59%. Biopsies were omitted in 96.8% of cases evaluated by colposcopists as negative, in 30.4% of cases evaluated as LG lesion, in 2.1% of cases evaluated as HG lesion, and in 0.3% of cases evaluated as neoplasia. Furthermore, it was observed that as the degree of the lesion increased, the number of biopsies consistently increased; more than one single biopsy was reported in 12.6% of cases with a colposcopic impression of LG, in 52.5% of cases of HG, and in 82.5% of cases with a colposcopic impression of cancer.

The correct site for performing biopsies was recognized in 58.9%, 77.3%, and 91.7% of histologically proven LG lesions (HPV-CIN1), HG lesions (CIN2-CIN3), and cervical cancer, respectively, while an incorrect site was indicated in 16.8%, 13.6%, and 5.3%. 

Noteworthy, non-biopsy rates accounted for 24.3% of HPV-CIN1 cases and for 12.1% of CIN2+ cases (*p* < 0.05) (Table 7).

Moreover, when the analysis focused on the subgroup of cases having a CIN2+ proven histology and a colposcopic impression of LG lesion expressed by colposcopists, the correctness of biopsy performance was significantly influenced by experience: junior colposcopists had a higher non-biopsy rate (20% vs. 10.1%), while seniors had a higher rate of correctly performed biopsies (73.9% vs. 66.9%) (*p* < 0.05) (Table 8).

## 4. Discussion

As colposcopy is a fundamental step as part of screening programs for the detection of pre-cancer cervical lesions, the success of the preventive strategy entirely depends on the diagnostic accuracy of the procedure. The assessment of colposcopy accuracy, in other words, the QC and QA processes, requires figures of the highest reliability in order to correctly evaluate the performance and effectiveness of colposcopic practice or to promote changes in standard requirements for operators.

This practical need deals with the objective issue of the very wide range of colposcopy accuracy figures available in the literature; meta-analysis studies have been published with the aim of providing statistically credible data to be used as comparison or reference values, thus allowing effective QC and QA processes in clinical practice. As an example, the most recently published meta-analysis, based on 15 studies and 22,764 cases, reports a combined sensitivity and specificity of 92% and 51% for a LG-SIL+ threshold and of 68% and 93%, respectively, for a HG-SIL+ threshold [18]. 

Unfortunately, data obtained in this fashion suffers from the significant bias of including papers with different study designs that influence the outcome reported; widely different figures are in fact reported depending on how the outcome of colposcopy is evaluated. Some studies investigate colposcopy outcome based upon the Colposcopic Impression (CI) that a CIN2+ is present; others evaluate the outcome on taking a biopsy because there is thought to be a Disease Present (DP), with the threshold of DP usually being a CIN1+. For this reason, the outcome measures have a significant effect on accuracy evaluation [19], indicating wide differences in both sensitivity and specificity.

That said, the present study, due to its main object of investigating and analyzing the performance of colposcopy mostly in terms of the QC of colposcopists and of the procedure, has to be seen as CI-based. Thereafter, the reported results are mainly discussed and compared with similar literature data. Nevertheless, some DP-based outcome assessments have been possible and are similarly discussed and compared.

The combined CI sensitivity and specificity (CIN2+ threshold) values obtained from the survey were 73.7% and 87.7%, respectively (see Table 1), with no statistically significant differences between senior and junior colposcopists; in general, this can be seen as a favorable result of the teaching programs of the involved institutions. These figures, compared with previous reviews [7,20], may be placed above weighted mean values for sensitivity and fully comparable with weighted mean values for specificity. Being the QC of Italian colposcopy/colposcopists the major objective of the study, these figures, together with the absence of significant differences between juniors and seniors, in our opinion, allow a more than satisfactory general evaluation of the colposcopy/colposcopists performance. The strength of this impression may further be supported considering the difficulty of the survey and the workload required of attendants.

This is particularly interesting in consideration of the experience level of the participants: since junior colposcopists performance accounted for better accuracy in each subset of thresholds, though without statistical significance, this may either reflect the good quality of the teaching programs in the institutions surveyed or the need for senior colposcopists to consider some kind of self-improvement. 

In terms of potential methodological biases, the use of static digital images of the cervix versus live colposcopy to assess the diagnostic accuracy and to perform QC evaluations, does not represent a limitation concerning the reliability of the sensitivity/specificity figures; as reported by Liu [21], recognitions of colposcopic patterns and colposcopic impression formulated on live colposcopy are reproducible on static digital images with high levels of agreement. Moreover, the use of a web-based program of digital colpophotographs, though with the different aim of assessing the accuracy of colposcopically directed biopsies, has already been proposed in Italy and demonstrated effective for QA purposes [9,22,23,24].

Regarding the results specifically directed to QC of colposcopists, we observed full agreement with the experts panel for the SCJ evaluation, following the 2011 IFCCP terminology [14], in 82.2% of *fully visible* SCJs, in 51.4% of *not fully visible* SCJs, and in 64.9% of *not visible* SCJs; in this analysis, a statistically significant difference was observed between seniors (67.5%) versus juniors (60.7%) for the *not visible* SCJ subgroup (*p* = 0.01). 

When SCJ was categorized following the 2017 ASCCP proposal [15], grouping the *not fully visible* and the *not visible* SCJ into one single category named *not fully visible*, full agreement with the experts increased to 75.4%, still having a statistically significant difference between seniors (77.1%) and juniors (72.8%) (*p* = 0.01).

Comparable comments can be made as far as it concerns the Transformation Zone (TZ): full agreement with the expert panel was achieved in 73.2%, 53.8%, and 66.7% of Type 1, Type 2, and Type 3 (2011 IFCPC terminology) [14], respectively; statistically significant differences were present between seniors and juniors for all three categories (see Table 4). The lowest rate of agreement for both SCJ visibility and the type of the TZ was recorded in the intermediate category.

Several authors have addressed the issue and the practical implications of adopting uniform and standardized colposcopy terminology, underlining the importance and accuracy improvement of the procedure when precise definitions of cervical patterns are widely utilized in clinical practice. In this view, the 2011 IFCPC terminology has represented a significant step forward in terms of colposcopy accuracy, having demonstrated better correlation with histology compared to traditional methods [25]. Despite that, the SCJ/TZ parameters have been repeatedly identified as the weak side of the process, as the intermediate categories, namely the *not fully visible* SCJ and the *Type 2* TZ, were always associated with the lowest grade of accuracy and reproducibility [26,27]. 

Our results consistently confirm this analysis and support the 2017 ASCPC proposal, detailing a significant increase in accuracy when a two-tailed classification of the SCJ is adopted, as recently published articles report [15,28].

The analysis of the grade of the TZ (G) and of the colposcopic impression compared with histology allows some comments that, in our opinion, are particularly interesting in terms of providing accuracy figures having both QC and QA meanings.

In terms of minor/major acetic acid alterations, full agreement was achieved in 76.25% (negative), 60.59% (G1), 59.11% (G2), and 64.64% (cancer suspicious). It is noteworthy that a *negative* interpretation and a *G1* interpretation underestimated 5.05% and 19.26% of CIN2+ histologically proven lesions, respectively (Table 5).

As far as it concerned the colposcopic impression, a *negative* impression and a *LG lesion* impression underestimated 3.06% and 21.2% of CIN2+ histologically proven lesions, respectively (Table 6). 

The analysis of these figures, performed consistently with the DP (CIN1+ threshold) principles of QA assessment, provided the following results: Overall, overrating the colposcopic impression was 1.5 times more common than underrating. However, when histologically proven HG lesions (CIN2-CIN3) were considered, overestimation and underestimation were fully comparable. It is in some way reassuring that only 3.06% of CIN2+ were considered colposcopically negative. Less reassuring is the detected 21% underestimation rate of CIN2+ lesions that were colposcopically interpreted as *LG lesions*. In terms of colposcopy principles, this should not represent a serious issue since an *LG lesion* colposcopic impression represents an indication for targeted biopsy, though the option of non-biopsy is acceptable [29]. Unfortunately, the balancing effect of the targeted biopsy in reducing the negative effect of colposcopic underestimation is largely influenced by real-life practice.

As shown in Table 7, our survey identified a 36.4% non-biopsy rate in histologically not-negative cases (24.3% of HPV-CIN1, 9.1% of CIN2-CIN3, and 3% of cancers, respectively). As reported, non-biopsy rates significantly decreased with increasing severity of histology (*p* < 0.05). These findings are interestingly consistent with several population-based studies on colposcopy QA [30,31]. Further, addressing the analysis specifically to cases with a *LG lesion* colposcopic impression and a CIN2+ histology, the non-biopsy rate accounted for 13.6%, with a statistically significant difference between seniors and juniors (10.1% vs. 20%) (*p* = 0.01) (Table 8). It clearly appears that experience in colposcopy plays an important role, significantly decreasing by 50% the risk of lower CI accuracy. 

In parallel, together with the non-biopsy rates, our figures regarding the correctness of biopsy-taking deserve some comments; correctly performed biopsies accounted for 58.9% of HPV-CIN1, 77.3% of CIN2-CIN3, and 91.7% of cancers. In our data, the overall amount of incorrect-site biopsies performed accounted for 16.8% in HPV-CIN1, 13.6% in CIN2-CIN3, and 5.3% in cancers (*p* < 0.05%); in the subgroup with an *LG lesion* colposcopic impression and CIN2+ histology, a biopsy was correctly performed in 71.4% of cases (seniors 73.9% vs. juniors 66.9%) (*p* < 0.05). 

As reported by Sideri [9], potential biases can be addressed when the accuracy of colposcopically targeted biopsy is investigated for QA purposes. Some may favor accuracy (e.g., the artificial conditions that may facilitate recognition of colposcopic features), while others may have the opposite effect (e.g., the impossibility of increasing the magnification and the single-shot chance given to participants). Nonetheless, the overall sensitivity does not appear to be significantly influenced by these factors.

Despite an overall good performance of the decision-making process for taking a colposcopically targeted biopsy, our results provide another confirmation that the sensitivity of biopsy for HG lesions is a justified concern; a large amount of data are available on the subject, consistently pointing to the need for improving options [5,32,33,34,35]. Colposcopists’ experience, though with marginal differences, has consistently been identified as positively influencing colposcopy accuracy [36,37].

Being cervical pre-cancer lesions detection the primary objective of colposcopy within cervical cancer screening programs, results from the present QC and QA assessments of colposcopy in Italy suggest some final considerations: (a) the overall sensitivity/specificity figures are in agreement with, and in some aspects better than, the mean figures reported by meta-analysis; (b) underestimation of colposcopy is particularly relevant when a *LG lesion* colposcopic impression is formulated; (c) the recommendation of taking a colposcopically targeted biopsy in cases of *LG lesion* colposcopic impression is justified by the rate of missed CIN2+ cases; (d) the low rate of statistically significant differences between experienced and junior colposcopists allows a favorable judgment of teaching programs; and (e) the need for continuous update, improvement, and QC of colposcopists is recommendable. In conclusion, the authors of the present article strongly believe that the adoption of colposcopy standards and quality recommendations by scientific societies is a fundamental step for effective cervical cancer prevention [10,11,12,13,29].

## Figures and Tables

**Table 1:** Diagnostic accuracy of colposcopy.

	Histology	All	Experience in Colposcopy	
			Seniors	Juniors
sensitivity	HPV or CIN 1	60.9%	56.8%	63.4%
	CIN2-CIN 3	63.9%	64.9%	62.3%
	Cancer	47.9%	47.3%	48.3%
	CIN 2+	73.7%	73.5%	73.9%
	overall	61.6%	60.6%	62.0%
specificity	Negative	77.1%	76.7%	77.4%
	HPV or CIN 1	87.7%	86.2%	88.6%

**Table 2:** SCJ assessment (2011 IFCPC terminology [14]).

Experts Panel	Colposcopists	*All*	Experience in Colposcopy		
			Seniors	Juniors	
fully visibile	**fully visibile ^#^**	**81.2%**	**80.3%**	**82.6%**	*p =* NS
	not fully visibile *	*12.9%*	*13.4%*	*12.1%*	
	*not visibile*	*5.9%*	*6.3%*	*5.3%*	
not fully visibile	*fully visibile*	*29.3%*	*28.2%*	*31.2%*	*p =* NS
	**not fully visibile**	**51.4%**	**51.9%**	**50.5%**	
	*not visibile*	*19.3%*	*19.9%*	*18.3%*	
not visibile	*fully visibile*	*15.2%*	*12.5%*	*19.6%*	*p =* 0.011
	*not fully visibile*	*19.9%*	*20%*	*19.7%*	
	**not visibile**	**64.9%**	**67.5%**	**60.7%**	

All colposcopists: *p* < 2.2^−16^; Cohen’s *kappa* correlation coefficient = 0.49 CI 95% [0.47–0.51]. Seniors: *p* < 2.2^−16^; Cohen’s *kappa* correlation coefficient = 0.49 CI 95% [0.47–0.52]. Juniors: *p* < 2.2^−16^; Cohen’s *kappa* correlation coefficient = 0.48 CI 95% [0.45–0.51]. ^#^ block letters = colposcopists vs. panel full agreement; * italics = incorrect SCJ judgment by colposcopists; SCJ = squamocolumnar junction; NS = not significant.

**Table 3:** SCJ assessment (ASCCP 2017 Nomenclature [15]).

Experts Panel	Colposcopists	All	Experience in Colposcopy		
			Seniors	Juniors	
fully visible	**fully visible ^#^**	**81.** **2%**	**80.** **3%**	**82.** **6%**	*p =* NS
	not fully visibile *	*18.8* *%*	*19.* *7%*	*17.* *4%*	
not fully visible	*fully visible*	*24.* *6%*	*22.* *9%*	*27.* *2%*	*p =* 0.011
	**not fully visibile**	**75.4%**	**77.1%**	**72.8%**	

All colposcopists: *p* < 2.2^−16^; Cohen’s *kappa* correlation coefficient = 0.57 CI 95% [0.54–0.59]. Seniors: *p* < 2.2^−16^; Cohen’s *kappa* correlation coefficient = 0.57 CI 95% [0.55–0.60]. Juniors: *p* < 2.2^−16^; Cohen’s *kappa* correlation coefficient = 0.56 CI 95% [0.52–0.59]. **^#^** block letters = colposcopists vs. panel full agreement; * italics = incorrect SCJ judgment by colposcopists; SCJ = squamocolumnar junction; NS = not significant.

**Table 4:** TZ assessment.

Experts Panel	Colposcopists	All	Experience in Colposcopy		
			Seniors	Juniors	
Type 1	**Type 1 ^#^**	**73.2%**	**69.5%**	**79%**	*p =* 1.029^−8^
	Type 2 *	*20.1%*	*22.3%*	*16.7%*	
	*Type 3*	*6.7%*	*8.2%*	*4.3%*	
Type 2	*Type 1*	*26.2%*	*23.7%*	*30%*	*p =* 7.006^−8^
	**Type 2**	**53.8%**	**52.3%**	**55.9%**	
	*Type 3*	*20%*	*24%*	*14.1%*	
Type 3	*Type 1*	*11.1%*	*9.1%*	*14.5%*	*p =* 7.58^−7^
	*Type 2*	*22.2 %*	*19.2%*	*27.2%*	
	**Type 3**	**66.7%**	**71.7%**	**58.3%**	

All colposcopists: *p* < 2.2^−16^; Cohen’s *kappa* correlation coefficient = 0.46 CI 95% [0.45–0.48]. Seniors: *p* < 2.2^−16^; Cohen’s *kappa* correlation coefficient = 0.46 CI 95% [0.44–0.48]. Juniors: *p* < 2.2^−16^; Cohen’s *kappa* correlation coefficient = 0.47 CI 95% [0.44–0.50]. **^#^** block letters = colposcopists vs. panel full agreement; * italics = incorrect SCJ judgment by colposcopists; TZ = Transformation Zone.

**Table 5:** Predictive value of colposcopic grade (G).

Colposcopic Grade	Histology			
	Negative	HPV or CIN 1	CIN2-CIN 3	Cancer
Negative	76.25% *	18.70%	4.21%	0.84%
G1	20.15%	**60.59%**	17.97%	1.29%
G2	4.70%	24.70%	**59.11%**	11.49%
Cancer	1.52%	3.05%	30.79%	**64.64%**

Pearson’s chi-squared test: *p* < 2.2^−16^; Cohen’s *kappa* correlation coefficient = 0.49 CI 95% [0.47–0.51]. * NPV; block letters = colposcopists vs. panel full agreement and PPV. G1 = minor colposcopic.

**Table 6:** Predictive value of colposcopic impression (CI).

Colposcopic Impression	Histology			
	Negative	HPV or CIN1	CIN2-CIN3	Cancer
Negative	77.9% *	18.5%	3%	0.6%
LG	18.8%	60%	19.7%	1.5%
HG	4.5%	25%	59.4% ^#^	11.1%
Cancer	1.2%	6.5%	27.9%	64.4% ^≈^

Pearson’s chi-squared test: *p* < 2.2^−16^; Cohen’s *kappa* correlation coefficient = 0.51—CI 95% [0.50–0.53]. * NPV = Negative Predictive Value; ^#^ PPV = Positive Predictive for CIN2-CIN3; ^≈^ PPV = Positive Predictive Value for cancer; LG = low-grade lesion; HG = high-grade lesion.

**Table 7:** Biopsy decision.

		Histology			
		Negative	HPV or CIN 1	CIN2-CIN 3	Cancer
biopsy	not performed	58.6%	24.3%	9.1%	3.0%
	yes, wrong site	1.7%	16.8%	13.6%	5.3%
	yes, correct site	39.7%	58.9%	77.3%	91.7%

Pearson’s chi-squared test: *p* < 2.2^−16^.

**Table 8:** Underestimation of colposcopic impression vs. biopsy decision.

	Biopsy	Experience in Colposcopy		
		All	Seniors	Juniors
LG Colposcopic Impression with CIN2+ histology	not performed	13.6%	10.1%	20%
	yes, wrong site	15%	16%	13.1%
	yes, correct site	71.4%	73.9%	66.9%
			*p* = 0.013	

LG = low-grade lesion.

## Data Availability

Research data are available on request from the corresponding author.
